# An investigation into “two hit” effects of BDNF deficiency and young-adult cannabinoid receptor stimulation on prepulse inhibition regulation and memory in mice

**DOI:** 10.3389/fnbeh.2013.00149

**Published:** 2013-10-21

**Authors:** Maren Klug, Maarten van den Buuse

**Affiliations:** ^1^Behavioural Neuroscience Laboratory, Mental Health Research InstituteMelbourne, VIC, Australia; ^2^Department of Psychology, Swinburne University of TechnologyHawthorn, VIC, Australia; ^3^Department of Pharmacology, University of MelbourneMelbourne, VIC, Australia

**Keywords:** brain-derived neurotrophic factor, prepulse inhibition, schizophrenia, cannabis, memory, mice

## Abstract

Reduced brain-derived neurotrophic factor (BDNF) signaling has been shown in the frontal cortex and hippocampus in schizophrenia. The aim of the present study was to investigate whether a BDNF deficit would modulate effects of chronic cannabis intake, a well-described risk factor for schizophrenia development. BDNF heterozygous mice (HET) and wild-type controls were chronically treated during weeks 6, 7, and 8 of life with the cannabinoid receptor agonist, CP55,940 (CP). After a 2-week delay, there were no CP-induced deficits in any of the groups in short-term spatial memory in a Y-maze task or novel object recognition memory. Baseline prepulse inhibition (PPI) was lower but average startle was increased in BDNF HET compared to wild-type controls. Acute CP administration before the PPI session caused a marked increase in PPI in male HET mice pre-treated with CP but not in any of the other male groups. In females, there were small increases of PPI in all groups upon acute CP administration. Acute CP administration furthermore reduced startle and this effect was greater in HET mice irrespective of chronic CP pre-treatment. Analysis of the levels of [^3^H]CP55,940 binding by autoradiography revealed a significant increase in the nucleus accumbens of male BDNF HET mice previously treated with CP but not in any of the other groups or in the caudate nucleus. These results show that BDNF deficiency and chronic young-adult cannabinoid receptor stimulation do not interact in this model on learning and memory later in life. In contrast, male “two hit” mice, but not females, were hypersensitive to the effect of acute CP on sensorimotor gating. These effects may be related to a selective increase of [^3^H]CP55,940 binding in the nucleus accumbens, reflecting up-regulation of CB1 receptor density in this region. These data could be of relevance to our understanding of differential “two hit” neurodevelopmental mechanisms in schizophrenia.

## Introduction

It is becoming widely accepted that the development of schizophrenia cannot be explained by single gene mutations or environmental factors. Instead, multiple factors, e.g., gene polymorphisms which increase risk, and environmental insults, such as stress or drug abuse, are likely to act synergistically to trigger disease onset, a model commonly referred to as the “two hit” hypothesis of schizophrenia (Bayer et al., 1999; Maynard et al., 2001; McGrath et al., 2003). Several animal model studies are attempting to delineate the molecular mechanisms involved in this synergism, with the ultimate aim to provide new treatment targets, including for early intervention. For example, studies have shown that temporary treatment with low doses of antipsychotics in late adolescence may be effective in preventing the emergence of schizophrenia-like behavioral phenotype in neurodevelopmental animal models of the illness (Piontkewitz et al., 2009). However, the mechanisms involved in such preventative treatments remain unclear.

Brain-derived neurotrophic factor (BDNF) is involved in brain development and neuroplasticity (Monteggia et al., 2004; Lu et al., 2008). Altered BDNF signaling has been implicated in a number of psychiatric illnesses, including schizophrenia and depression (Angelucci et al., 2005; Autry and Monteggia, 2012). For example, post-mortem studies have found significant reductions of BDNF gene expression and protein levels in the brains of people with schizophrenia (Hashimoto et al., 2003; Weickert et al., 2003; Durany and Thome, 2004). Levels of BDNF in the brain are modulated by stress in an age-, sex-, and stress-type-dependent manner (Bath et al., 2013). We have previously used chronic administration of the stress hormone, corticosterone, as a model of late adolescent/early adulthood stress. Corticosterone treatment in BDNF heterozygous (HET) mice caused sex-specific long-term effects in the Y-maze and other effects (Klug et al., 2012).

Cannabis abuse may precipitate psychosis development in vulnerable individuals, much like a “two hit” effect (Van Os et al., 2002; Henquet et al., 2008; Gururajan et al., 2012). BDNF may be part of the neurochemical mechanism involved in this interaction. Previous studies have shown that cannabis use can alter levels of BDNF in humans as well as in animals (Derkinderen et al., 2003; Jockers-Scherübl et al., 2004; Butovsky et al., 2005; D'souza et al., 2009). For example, injection of Δ^9^-tetrahydrocannabinol (THC) has been shown to upregulate BDNF expression in rats and mice (Derkinderen et al., 2003; Butovsky et al., 2005) and BDNF serum levels in healthy humans (D'souza et al., 2009). However, in human studies looking at chronic abuse of cannabis, it is not as clear in which direction cannabis exerts its influence on BDNF. While some have shown that chronic cannabis users have lower basal levels of serum BDNF (D'souza et al., 2009) others could not detect such differences (Jockers-Scherübl et al., 2004; Angelucci et al., 2008). In a study focusing on the val66met BDNF polymorphism, in female psychotic patients, cannabis use was associated with a 7 year-earlier onset of the disease when patients were BDNF-Met carriers but not when they were Val/Val genotypes (Decoster et al., 2011). These results suggest interactions between cannabis use, BDNF and psychotic disorders, even though the mechanism involved remains unclear. We recently investigated maternal separation in rats as a first developmental “hit” and treated the animals with the cannabinoid receptor agonist, CP55,940 (CP) (Klug and Van Den Buuse, 2012). Male “two hit” rats, but not female rats, showed anhedonia-like behavior and increased anxiety, but no deficits in short-term spatial memory (Klug and Van Den Buuse, 2012). Thus, treatment with CP in maternally-separated rats caused markedly different long-term effects than those seen in BDNF HET mice treated with corticosterone. This suggested marked qualitative differences in the two hit effects of either stress (in the form of corticosterone treatment) compared to cannabis abuse (in the form of chronic CP injections). However, species differences could not be ruled out. Therefore, the present study is focused on combining genetically-induced deficiency of BDNF in mice and treatment with the cannabinoid receptor agonist, CP, during adolescence/young adulthood.

We used BDNF heterozygous (HET) mice and treated them with CP from 6 to 9 weeks of age (adolescence/young adulthood) which represents a critical time window for detrimental effects of cannabis abuse (Schneider, 2008). We investigated locomotor activity at the beginning and end of treatment, as well as 2 weeks after treatment had ceased. To investigate whether cognition was affected in the animals, we used the Y-maze and the novel object recognition test. Baseline PPI was compared between the groups and, in addition, in a separate session the mice received an acute injection of CP to see whether differences in reaction to the drug would occur between animals previously treated with the cannabinoid agonist and animals previously treated with vehicle.

## Methods

### Animals and protocols

Male and female BDNF heterozygous and wild-type control mice were obtained from a breeding colony at the Florey Neuroscience Institutes animal facility and brought over to the Mental Health Research Institute at 4–5 weeks of age, where they were housed in individually-ventilated cages (IVC). All experiments and procedures were approved by the Animal Experimentation Ethics Committee of the Florey Neuroscience Institutes, University of Melbourne, Australia.

The mice received injections with 0.4 mg/kg CP55,940 or vehicle from 6 to 9 weeks of age. CP55,940 ((-)-cis-3-[2-hydroxy-4-(1,1,-dimethylheptyl)phenyl]-trans-4-(3-hydroxypropyl) cyclohexanol) was obtained from Tocris Bioscience (Bristol, UK) and first dissolved in 100% ethanol and then diluted in Tween 80 and saline to make a final vehicle solution of 2.5% ethanol, 2.5% Tween 80, and 95% saline. Mice were injected intraperitoneally (i.p.) once a day (at 9:00 am) on weekdays at a dose of 0.4 mg/kg in a volume of 10 ml/kg. The treatment started at 6 weeks of age and continued for 3 weeks until the mice were 9 weeks of age. The mice received no injections on the weekends. Control animals received vehicle solution injections. Thus, there were four male and four female experimental groups: wild-type mice treated with vehicle (WT/veh), wild-type mice treated with CP55,940 (WT/CP), BDNF heterozygous mice treated with vehicle (HET/veh) and BDNF HET mice treated with CP55,940 (HET/CP). Each group consisted of 10–16 animals.

Body weights were obtained at 6 weeks of age (CP treatment start), 9 weeks of age (end of CP treatment) and 12 weeks of age (during behavioral testing). Behavioral testing started at 11 weeks of age and included locomotor activity, Y-maze, novel object recognition, and prepulse inhibition (PPI).

### Locomotor activity

To assess locomotor hyperactivity, mice were placed into individual automated photobeam activity cages (Tru Scan, Coulbourn Instruments, Whitehall, PA, USA; 27 cm l × 27 cm w × 40 cm h). Each cage was fitted out with photobeams which detected an animal's position at any one time during a session. The associated software used this information to calculate the distance moved in centimetres by the animal in 5 min intervals. Mice were placed into the chambers for 60 min to habituate them to the new environment and record baseline activity. Each animal was tested three times for changes in locomotor activity: at the beginning of CP treatment (6 weeks of age), at the end of the treatment (9 weeks of age) and 2 weeks after treatment had ceased (11 weeks of age). During the first two sessions, the mice were placed into the chambers immediately after injection with the daily dose of CP or vehicle and total distance moved was recorded for 12 five min blocks. At 11 weeks of age, locomotor activity was similarly measured but the animals received no injection.

### Y-maze

Short-term spatial memory was assessed using the Y-maze (Dellu et al., 1992, 2000), which consisted of three arms (30 cm l × 8 cm w × 16 cm h) with geometric shapes on the wall and a triangular central platform. In the first exposure session, animals were placed facing the wall at the end of one arm (start arm) and allowed to explore the Y-maze for 10 min with one of the two other arms closed-off (novel arm). After a retention period, animals were placed back into the start arm and allowed to explore all three arms of the maze for 5 min. Behavior was recorded on video and analyzed with video tracking software (Ethovision, Noldus, The Netherlands). Behaviors analyzed were time spent in each arm, percentage number of entries to each arm and the overall number of arm entries to measure locomotor activity. We chose to display percentage number of entries to account for possible differences in locomotor activity. Mice were tested twice in the Y-maze with two different retention periods of 1 or 2 h (Dellu et al., 1992, 2000; Conrad et al., 2003) but because similar results were obtained, only data pertaining to the 2-h interval will be shown here.

### Novel object recognition

Object recognition was assessed using the novel object recognition task. Two days before testing, animals were habituated to the empty testing apparatus (40 cm l × 30 cm w × 10 cm h) for 10 min each day. On the third day the animals were tested for novel object recognition memory. On the testing day, two identical objects were placed in the right and left corner at the end of the arena. The animals were then placed into the arena facing the opposite wall and were allowed to explore the objects for 10 min (introduction phase). The animals were then removed and returned back to their home cages for 2 h. Following the delay, the animals were placed back into the arena, which now contained one familiar object (from the introduction phase) and one novel object. The animals were allowed to explore the objects for 5 min (recognition phase). Behavior in both phases was recorded on video and analyzed with video tracking software (TopScan, CleverSys Inc., Reston, VA, USA). The time spent sniffing the objects in the introduction phase was analyzed and used as a measure to detect side preference and interaction time. The time spent sniffing the objects in the recognition phase was used as a measure of recognition. Animals that spent less than 10 s exploring both objects were excluded from the analysis. The amount of time spent investigating the novel object was expressed as percentage of total object exploration time.

### Prepulse inhibition of acoustic startle

PPI was measured using eight automated startle chambers (SR-LAB, San Diego Instruments, San Diego, CA, USA). Animals were placed individually in a Plexiglas cylinder of 3.8 cm diameter which was secured to a platform with a piezoelectric accelerometer mounted under it to detect whole body startle responses. The cylinders were placed in a ventilated, sound-attenuated and well-lit startle box. Background noise and acoustic pulses were presented through a speaker in the startle box and responses were measured with the SR-Lab software (San Diego instruments) running on a computer in an adjacent room.

A single PPI session included 104 trials and lasted ~40 min. There was an initial 3 min acclimation period with continuous background white noise only, set at 70 dB, which continued throughout the rest of the entire session. The first and last eight trials were presented at 115 dB. The 88 trials in-between were presented in a pseudo-random order and included sixteen 115 dB startle pulses and 72 prepulse-pulse trials including eight of each prepulse (PP) intensity of 2, 4, 8, 16 dB over baseline and eight “no-stimulus” trials, where no pulse was presented. Each startle pulse was 40 ms in duration, the prepulse was 20 ms and there were either a 30 or 100 ms inter-stimulus interval (ISI) between the prepulse and the pulse. Because the clearest group differences were observed with the 100 ms ISI, only those data will be shown here. Startle amplitude was analyzed as the average of all 115 dB pulse-alone trials. Startle habituation was analyzed by using the pulse-alone data in four blocks of eight trials. PPI was calculated as the percentage difference of the responses to pulse-alone trials minus prepulse trials divided by the response to pulse-alone trials.

The PPI experiment included a saline treatment session and a challenge session with an acute injection of CP (0.4 mg/kg). Saline and CP were administered in a volume of 10 ml/kg, 5 min prior to the PPI session. There were 3 days of drug wash-out in-between the two test sessions. The order of drug administration was randomized to control for the possibility of test habituation.

One week after the last behavioral test, mice were killed by cervical dislocation and the brains stored at −80°C until further use.

### [^3^H]CP55,940 binding

The procedure was essentially as previously described (Chavez et al., 2010) with modifications. Briefly, 20 μm brain sections including the cingulate cortex, caudate nucleus, and nucleus accumbens were cut on a cryostat and thaw-mounted on gelatinized slides. On the day of the experiments, the slides were first washed for 30 min in a 50 mM Tris HCl buffer with 1% BSA (pH 7.4) at room temperature. The sections were then exposed to either the total binding solution, containing [^3^H]CP55,940 at a final concentration of 2 nM, or the non-specific binding solution containing [^3^H]CP55,940 and 10 μM of CP55,940 for 2 h at room temperature. The reaction was terminated by three 20 min washes in ice cold buffer. The sections were then air-dried and partially fixed in paraformaldehyde vapor overnight before being placed in Fujifilm BAS 2025 cassettes (Berthold, Australia) and apposed to BAS-TR2025 phosphoimaging plates (Berthold) for 6 days. Autoradiographic images were analyzed using AIS image analysis software (Imaging Research, ON, Canada). Binding densities in the nucleus accumbens and caudate nucleus were quantitated by comparing them to [^3^H] microscales and expressed as fmol/mg estimated tissue equivalent (ETE) (Pavey et al., 2002; Chavez et al., 2010). It should be noted that this single-point agonist binding method most likely represents high-affinity binding and does not distinguish between possible changes in Kd or Bmax of the receptor.

### Data analysis

Data were expressed as the mean ± the standard error of the mean (SEM). Data analysis was conducted with the software program SPSS Statistics GradPack 17.0 (SPSS Inc, Chicago, IL, USA). All data were analyzed using a Three-Way analysis of variance (ANOVA) with repeated measures where appropriate, with factors being sex, first “hit” (BDNF heterozygosity) and second “hit” (CP) as main factors. When interactions were found, separate Two-Way ANOVAs split by one of the independent factors or pairwise comparisons of appropriate group combinations were done to clarify the results (Nieuwenhuis et al., 2011). If *P* < 0.05, differences were considered statistically significant.

## Results

### Body weight

At 6 weeks of age (start of drug treatment) female mice weighed significantly less than male mice [main effect of sex: *F*_(1, 98)_ = 135.2; *P* < 0.001] and HET mice had higher bodyweight compared to their wild-type littermates [main effect of genotype: *F*_(1, 98)_ = 11.7; *P* = 0.001]. At 9 weeks of age (end of drug treatment) statistical analysis revealed a number of main effects [main effect of sex: *F*_(1, 98)_ = 290.7; *P* < 0.001; main effect of genotype: *F*_(1, 98)_ = 38.9; *P* < 0.001 and main effect of CP treatment: *F*_(1, 98)_ = 13.2; *P* < 0.001] and also a significant interaction for sex × genotype [*F*_(1, 98)_ = 4.8; *P* = 0.041]. Two-Way ANOVAs split by sex revealed that male HET mice weighed more compared to male wild-type mice [main effect of genotype: *F*_(1, 49)_ = 8.2; *P* = 0.006] and CP-treated animals had lower bodyweight compared to vehicle-treated mice independent of genotype [main effect of CP treatment: *F*_(1, 49)_ = 10.7; *P* = 0.002]. For female mice there was a significant main effect of genotype with HET mice having higher bodyweights [*F*_(1, 48)_ = 36.7; *P* < 0.001]. At 12 weeks of age (during behavioral testing) the treatment effect of CP had disappeared but a significant genotype effect remained for both males [*F*_(1, 49)_ = 11.3; *P* = 0.002] and females [*F*_(1, 48)_ = 42.2; *P* < 0.001] and with HET mice weighing more than wild-types (Table 1).

**Table 1:** **Bodyweight (grams) of wild-type (WT) and BDNF heterozygous (HET) mice treated with vehicle (veh) or CP55,940 (CP) at 6 weeks (beginning of drug treatment), 9 weeks (end of drug treatment), and 12 weeks of age (during behavioral testing)**.

	**WT/veh**	**WT/CP**	**HET/veh**	**HET/CP**
**MALE MICE**				
Week 6	21.4 ± 0.4	20.7 ± 0.5	21.9 ± 0.3	21.9 ± 0.5
Week 9	24.3 ± 0.5	22.7 ± 0.3	25.3 ± 0.4	24.1 ± 0.4
Week 12	26.4 ± 0.7	26.0 ± 0.5	28.6 ± 0.5	27.7 ± 0.6
**FEMALE MICE**				
Week 6	16.6 ± 0.5	16.8 ± 0.2	18.5 ± 0.4	17.9 ± 0.6
Week 9	18.2 ± 0.4	17.9 ± 0.2	21.0 ± 0.6	19.8 ± 0.4
Week 12	19.7 ± 0.5	20.0 ± 0.3	24.7 ± 1.0	23.1 ± 0.6

Data are mean ± standard error of the mean (SEM) of 10–16 animals per group. At all ages, male mice weighed significantly more than female mice and HET mice weighed more than their wild-type littermates. For further details on statistical analysis, see text.

### Locomotor activity

**Figure 1:**
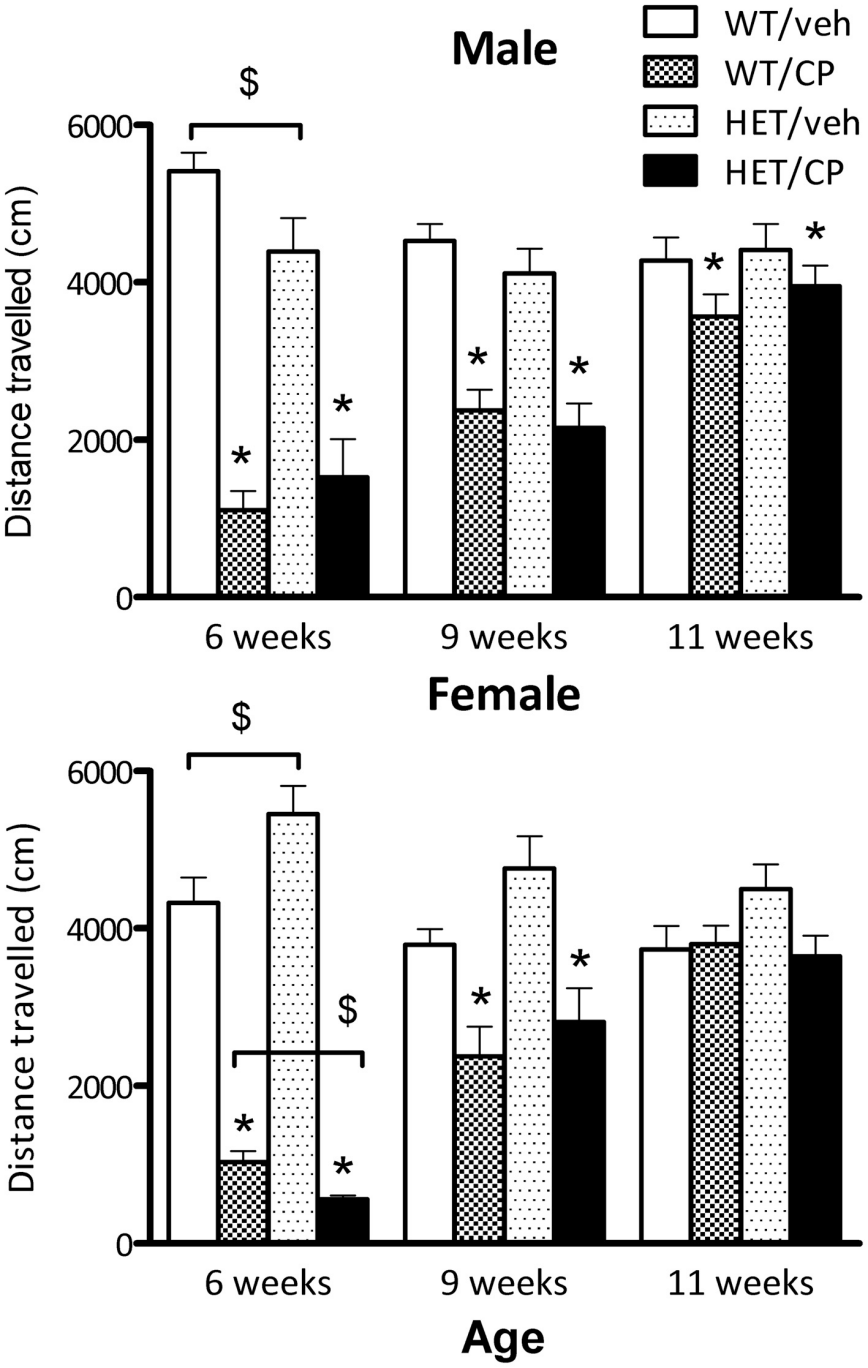
**Total distance traveled (cm) by male and female mice in automated photobeam cages at the start of CP55,940 (CP) treatment at 6 weeks of age, end of CP treatment at 9 weeks of age and 2 weeks after treatment had ceased.** Wild-type and HET mice were either treated with vehicle solution or CP from 6 to 9 weeks of age. CP treatment significantly reduced distance traveled in the first and second session in both sexes and distance traveled was still slightly, but significantly reduced in male mice 2 weeks after treatment had ceased (for statistical analysis see text). Additional effects between groups of animals analyzed with pairwise comparison are indicated in the figure: ^*^P < 0.05 for differences between vehicle-treated controls and CP-treated mice based on main effect of CP in ANOVA. ^$^*P* < 0.05 for difference between genotypes based on pair-wise *post-hoc* ANOVAs.

At the beginning of the CP treatment period at 6 weeks of age, both sexes showed significantly reduced distance moved after the injection compared to vehicle-injected controls [main effect of CP: *F*_(1, 46)_ = 109.4 for male mice and *F*_(1, 43)_ = 207.0 for female mice, both *P* < 0.001]. Additionally, in both sexes a significant interaction for genotype × CP emerged [males: *F*_(1, 46)_ = 4.4; *P* = 0.042; females: *F*_(1, 43)_ = 7.8; *P* = 0.008]. For male mice, pairwise comparisons revealed that HETs were less active compared to wild-type mice when treated with vehicle [*F*_(1, 23)_ = 4.8; *P* = 0.040] but there were no differences after CP treatment (Figure 1). For female mice, pairwise comparisons revealed that HETs were more active compared to wild-type controls [*F*_(1, 24)_ = 5.4; *P* = 0.029]. However, when the mice had received CP injection, this phenotype was reversed with HET mice being less active compared to wild-types [*F*_(1, 18)_ = 9.5; *P* = 0.007].

At the end of CP treatment at 9 weeks of age, both male and female mice still showed acute hypo-activity after CP injection compared to saline-injected controls [main effect of CP: *F*_(1, 50)_ = 24.5 and *F*_(1, 48)_ = 22.5 for male and female mice, respectively, both *P* < 0.001] although the extent of the effect appeared smaller than at 6 weeks of age at the start of the CP treatment (Figure 1). There were no significant differences between wild-type mice and HET mice (Figure 1).

Two weeks after treatment had ceased and animals were tested for baseline locomotor activity without drug injections, male wild-type and HET mice treated previously with CP showed a strong trend for reduced distance traveled [*F*_(1, 50)_ = 4.0; *P* = 0.051]. In contrast, in female mice no main effects or interactions were found (Figure 1).

### Y-maze

**Figure 2:**
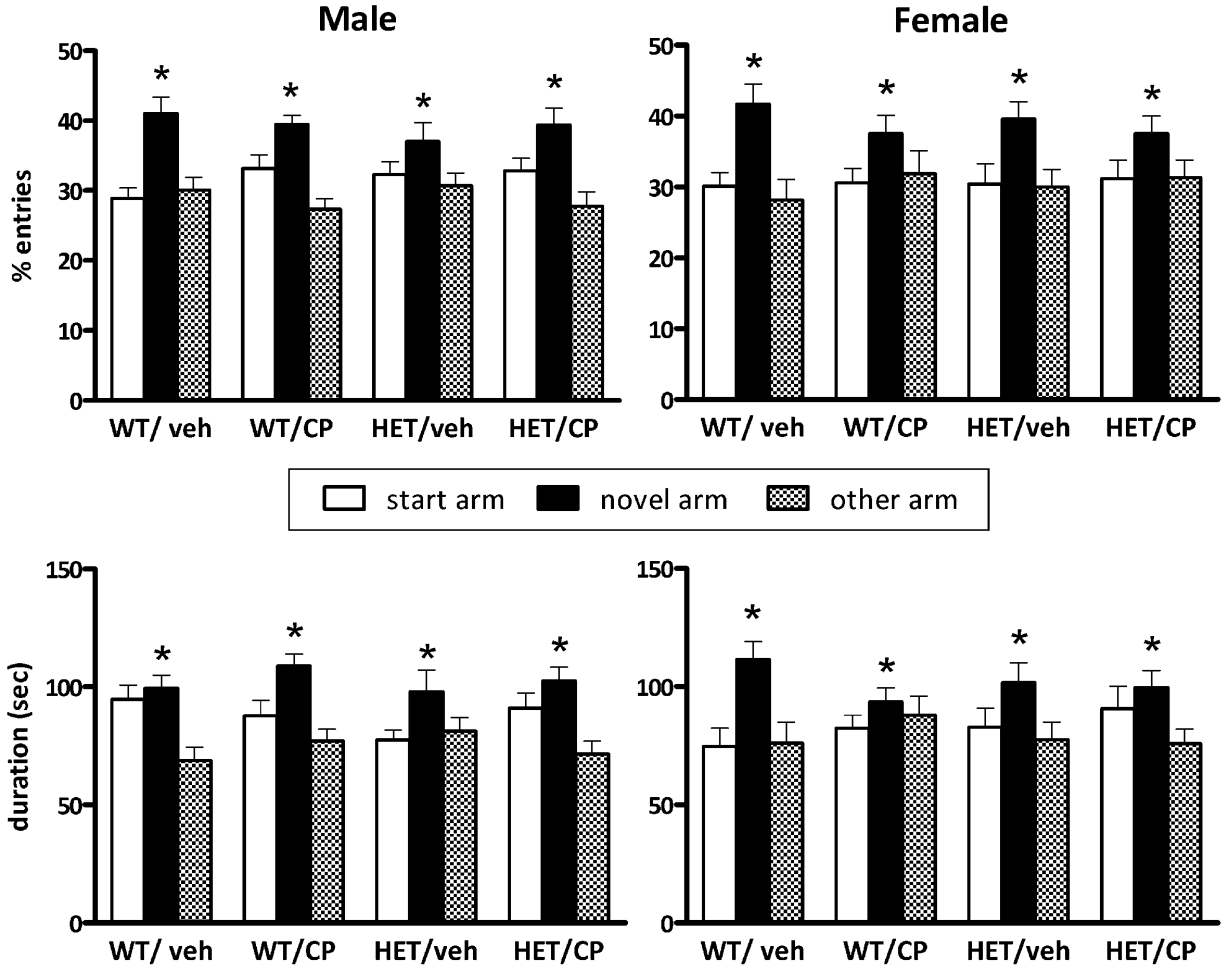
**Y-maze behavior after a 2 h retention period of BDNF heterozygous (HET) and wild-type (WT) mice treated with vehicle (veh) or CP55,940 (CP).** (**Top**) show the percentage number of entries into the start, novel and “other” arm of the Y-maze during the 5 min re-exposure session and the (**Bottom**) show the duration of time spent in each arm. Data are presented for male (**Left**) and female mice (**Right**) and are expressed as mean ± SEM. ^*^P < 0.05 for difference between novel arm, start arm, and “other” arm based on ANOVA. All groups showed significant preference for the novel arm and there were no effects of genotype or CP treatment.

After a 2 h retention period all animals appeared to be able to remember their previous encounter with the Y-maze indicated by a similar preference in the percentage of number of visits to the novel arm [*F*_(2, 82)_ = 22.4; *P* < 0.001, no interactions] and the most time spent in the novel arm [*F*_(2, 82)_ = 20.8; *P* < 0.001, no interactions] (Figure 2). The total number of arm entries differed significantly between genotypes [*F*_(1, 90)_ = 4.9; *P* = 0.030] with HETs having a higher number of arm entries indicating higher general locomotor activity (Table 2).

**Table 2:** **Total number of arm entries in the Y-maze after a 2-h retention period**.

	**WT/veh**	**WT/CP**	**HET/veh**	**HET/CP**
Male mice	35.5 ± 2.3	36.7 ± 4.2	35.6 ± 2.3	42.3 ± 3.4
Female mice	39.1 ± 3.3	32.9 ± 2.7	41.6 ± 3.1	40.7 ± 1.5

Data are expressed as mean ± SEM. HET mice had a higher number of arm entries. For statistical analysis, see text.

### Novel object recognition

**Figure 3:**
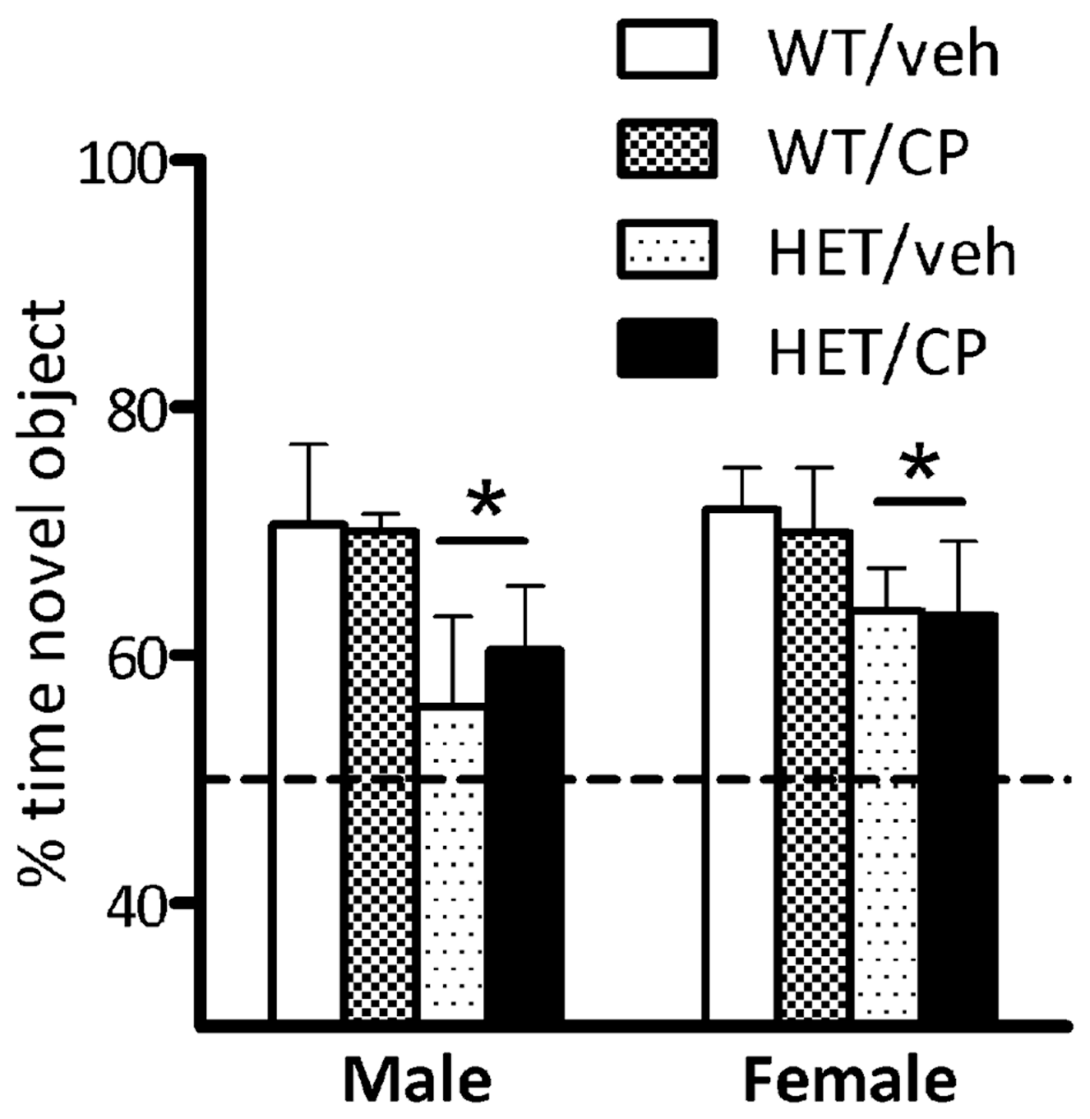
**Percentage of time exploring the novel object in the recognition phase of the novel object recognition test.** Data are for male and female wild-type (WT) and BDNF heterozygous mice (HET) treated with vehicle (veh) or CP55,940 (CP) and are expressed as mean ± SEM. ^*^P < 0.05 for difference between HET mice and wild-type controls based on ANOVA main effect. HET mice spent less time with the novel object compared to wild-type controls but there was no effect of young-adult CP treatment.

During the introduction phase there were no differences in the amount of time any of the groups spent investigating the two objects (data not shown). During the recognition phase, analysis of the percentage of time spent with the novel object revealed a main effect of genotype [*F*_(1, 73)_ = 8.2; *P* = 0.006] but no other main effects or interactions. This reflects that HET mice spent less time with the novel object compared to wild-type controls indicating poorer object recognition memory in this genotype. Inspection of the data (Figure 3) suggests that this was particularly prominent in male mice but there was no main effect of sex of the animals nor a genotype × sex interaction.

### Prepulse inhibition of acoustic startle

Combined analysis of PPI in male and female mice revealed significant main effects of sex of the animals [*F*_(1, 88)_ = 5.0; *P* = 0.028], genotype [*F*_(1, 88)_ = 23.8; *P* < 0.001], acute CP treatment [*F*_(1, 88)_ = 22.4; *P* < 0.001] and an interaction of acute CP × sex × prepulse intensity [*F*_(3, 264)_ = 3.8; *P* = 0.010].

**Figure 4:**
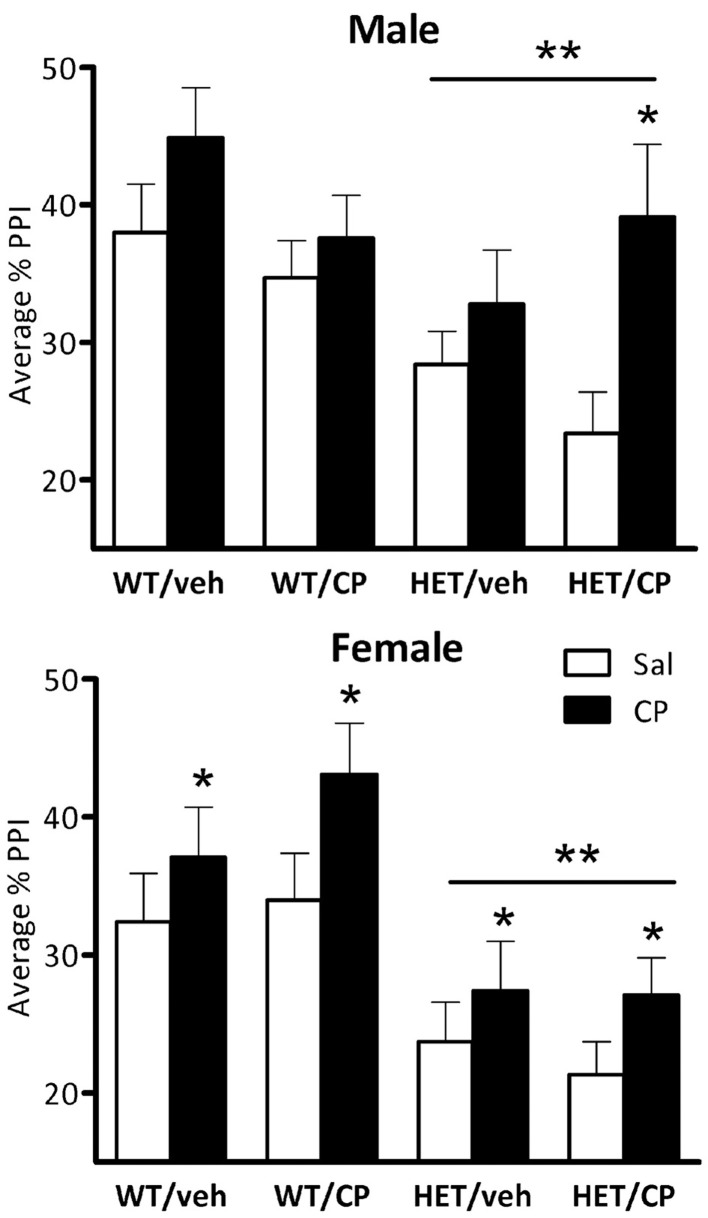
**Average percentage PPI in the four treatment groups: WT/veh, WT/CP, HET/veh, and HET/CP in both sexes.** All groups were administered saline (Sal; open bars) and 0.4 mg/kg CP55,940 (CP; black bars) before the PPI test. HET mice had significantly lower PPI after saline injection compared to wild-type controls. Acute CP treatment increased PPI in male “two hit” mice and in all female groups. ^*^P < 0.05 for difference with acute saline based on genotype × acute treatment interaction and *post-hoc* comparisons (males) or main ANOVA effect (females). ^**^P < 0.05 for difference between HET mice and wild-type controls based on ANOVA main effect.

**Figure 5:**
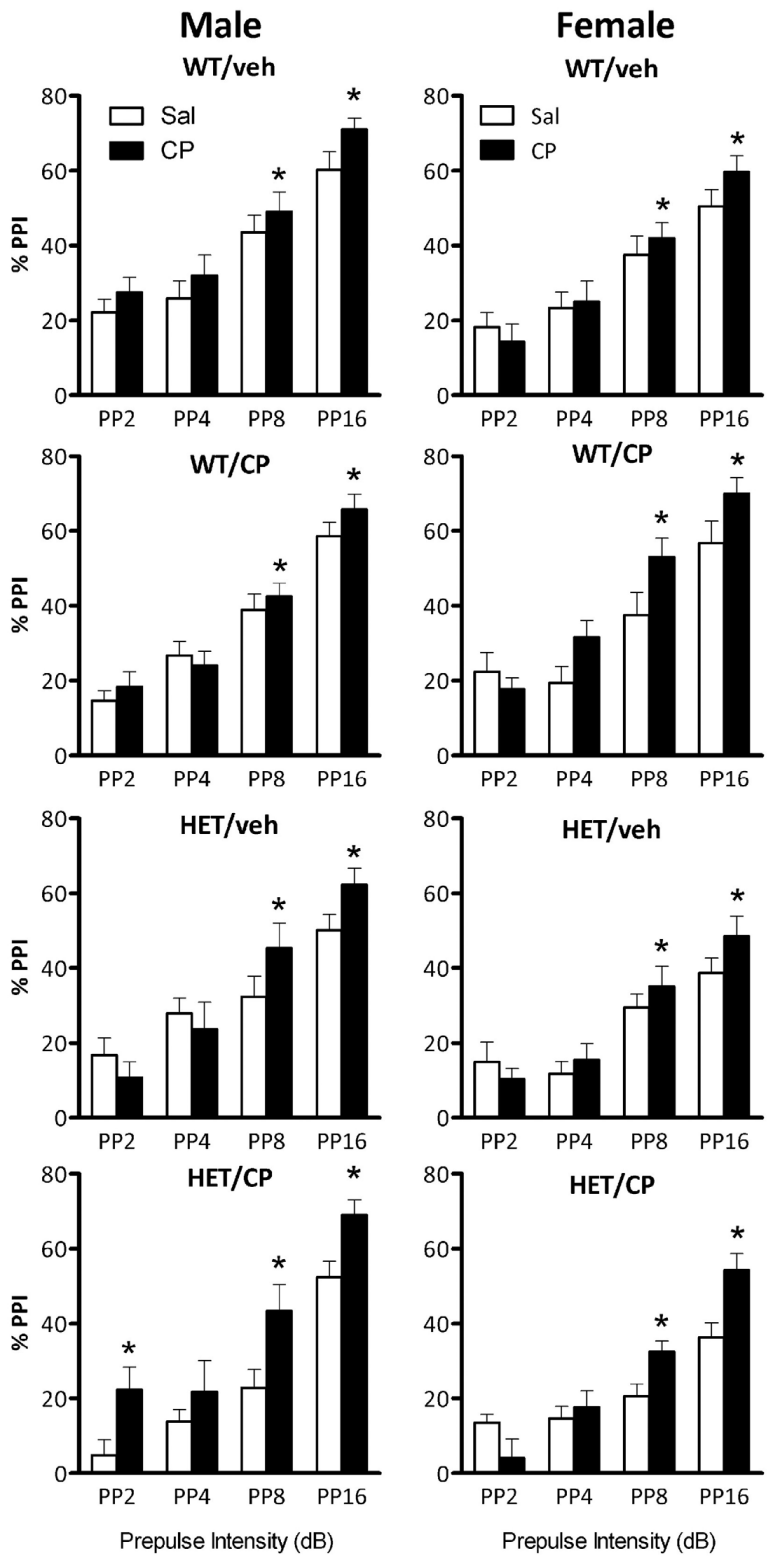
**Percentage prepulse inhibition (PPI) expressed per prepulse intensity in the four treatment groups: WT/veh, WT/CP, HET/veh, and HET/CP in both sexes.** All groups were administered saline (Sal; open bars) or 0.4 mg/kg CP55,940 (CP; black bars) PP2, PP4, PP8, and PP16 indicate prepulse intensities of 2, 4, 8, and 16 dB over the 70 dB background with a 100 ms interstimulus interval. Data are mean ± SEM. ^*^P < 0.05 for difference with acute saline treatment based on ANOVA main effect (PP8 and PP16) or CP × genotype × pre-treatment interaction and pair-wise comparison (PP2).

In male mice, ANOVA revealed a main effect of genotype [*F*_(1, 46)_ = 7.3; *P* = 0.010], reflecting that PPI was generally lower in HET compared to wild-type mice (Figure 4). Acute CP treatment increased PPI [*F*_(1, 46)_ = 14.1; *P* < 0.001] and this effect was different depending on the prepulse intensity [*F*_(3, 138)_ = 4.8; *P* = 0.003, Figure 5] and tended to interact with genotype and young-adult pre-treatment [*F*_(1, 46)_ = 3.7; *P* = 0.059]. Analysis for each prepulse intensity separately revealed that at PP8 and PP16 all groups showed a significant increase in PPI upon acute CP injection [main effects *F*_(1, 46)_ = 15.3 and 28.7, respectively, both *P* < 0.001, no interactions]. In contrast, analysis of PP2 revealed a CP × genotype × pre-treatment interaction [*F*_(1, 46)_ = 5.2; *P* = 0.028] and further analysis showed a marked increase in PPI at the PP2 intensity [*F*_(1, 10)_ = 8.1; *P* = 0.017] in HET mice pre-treated with CP but not in any of the other groups (Figure 5). There were no significant effects at PP4.

Inspection of the data (Figures 4, 5) revealed that HET mice pre-treated in young-adulthood with CP had the lowest baseline PPI of all groups which could explain their larger response to subsequent acute CP treatment. However, further analysis in the four groups after acute injection of saline only confirmed the generally lower PPI in HET mice [main effect of genotype: *F*_(1, 46)_ = 11.7; *P* = 0.001] independent of adolescent CP pre-treatment (Figure 4). Interestingly, analysis of data obtained after acute CP injection showed no genotype effect, suggesting normalization of PPI in the HET mice by acute CP injection (Figure 4).

Also in female mice PPI was lower in HET compared to wild-type controls [*F*_(1, 42)_ = 18.4; *P* < 0.001, Figure 4]. Acute CP treatment increased PPI [*F*_(1, 42)_ = 8.7; *P* = 0.005, Figure 4] and, similar to male mice, this effect was greatest at higher prepulse intensities [CP × prepulse interaction: *F*_(3, 126)_ = 11.7; *P* < 0.001, Figure 5]. Further analysis for each prepulse intensity confirmed this observation as a significant main effect of acute CP treatment was found in all groups at PP8 and PP16 [*F*_(1, 42)_ = 14.5 and 20.4, respectively, both *P* < 0.001, no interactions] but not PP4 or, in contrast to male mice, PP2 (Figure 5).

### Startle responses

**Figure 6:**
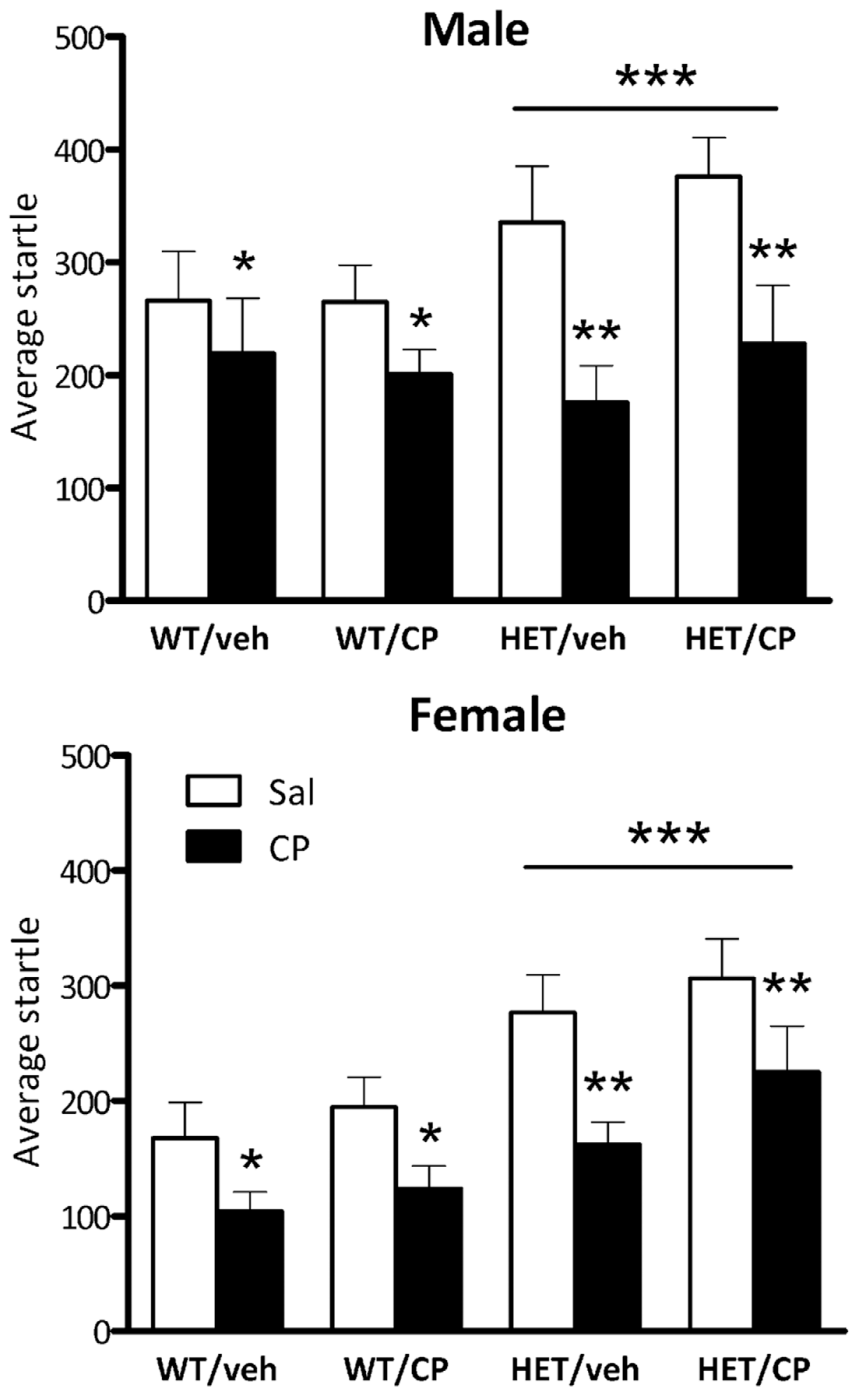
**Average startle amplitude in the four treatment groups: WT/veh, WT/CP, HET/veh, and HET/CP in both sexes.** All groups were administered saline (Sal; open bars) and 0.4 mg/kg CP55,940 (CP; black bars). Data are mean ± SEM. ^*^P < 0.05 for difference with saline based on ANOVA main effect. ^**^P < 0.05 for greater effect of acute CP injection in HET mice based on ANOVA CP × genotype interaction. ^***^P < 0.05 for difference in startle amplitudes between WT and HET mice based on ANOVA main effect.

Average baseline startle amplitude was significantly lower in female mice than in male mice [*F*_(1, 87)_ = 8.9; *P* = 0.004] and HET mice had higher startle compared to wild-type mice independent of the sex of the animals [*F*_(1, 97)_ = 14.4; *P* < 0.001; Figure 6]. Acute CP injection significantly decreased average startle [*F*_(1, 87)_ = 56.7; *P* < 0.001], an effect which was greater in HET mice [CP × genotype interaction: *F*_(1, 87)_ = 6.9; *P* = 0.010] but was independent of the sex of the animals. However, when analysis was split up by genotype both groups were significantly affected by acute CP injection ([*F*_(1, 50)_ = 13.6; *P* = 0.001 and *F*_(1, 39)_ = 41.9; *P* < 0.001] for wild-type and HET mice, respectively, Figure 6). Notably, the acute effect of CP on startle was not affected by chronic young-adult CP treatment in any of the groups (Figure 6).

**Figure 7:**
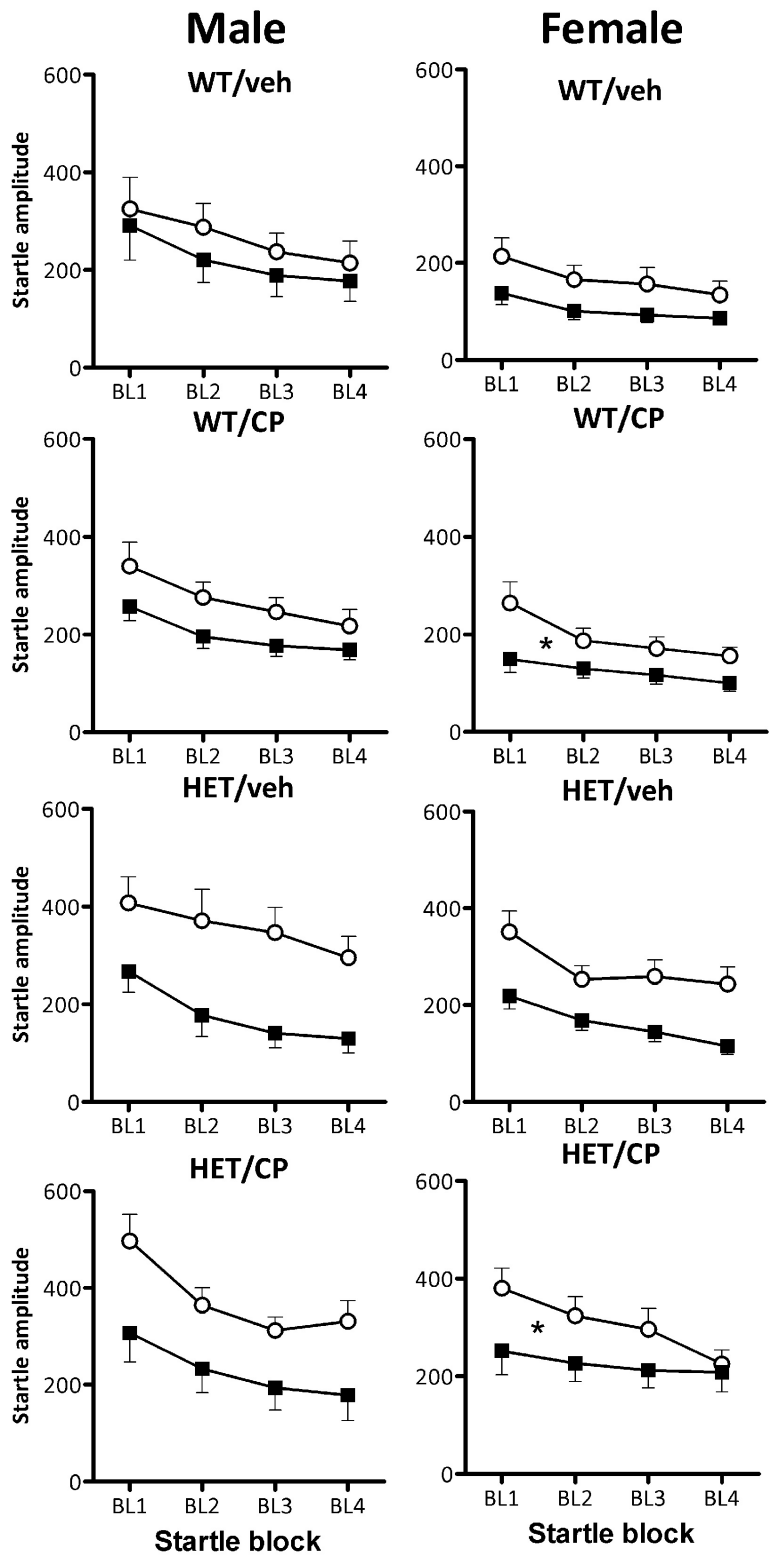
**Startle habituation expressed per startle block in the four treatment groups: WT/veh, WT/CP, HET/veh, and HET/CP in both sexes.** All groups were acutely administered saline (Sal; open circles) and 0.4 mg/kg CP55,940 (CP; black squares). Data are mean ± SEM. ^*^P < 0.05 for acute CP injection to reduce startle habituation in female WT and HET mice previously treated with CP in young-adulthood based on ANOVA interaction. Even though acute CP injection reduced startle overall (see Figure 6), it had no effect on startle habituation in any of the other groups. For statistical analysis, see text.

Startle habituation occurred in all groups as indicated by a main effect of block [*F*_(3, 261)_ = 104.2; *P* < 0.001]. Overall, female mice showed less startle habituation than male mice [*F*_(3, 261)_ = 4.1; *P* = 0.008] but there was no difference between the genotypes in terms of startle habituation (Figure 7). Acute CP treatment reduced startle habituation in a manner dependent on young-adult CP pre-treatment [*F*_(3, 261)_ = 2.9; *P* = 0.037]. In male mice, there were no effects of acute CP injection on startle habituation (Figure 7). In contrast, in female mice, acute CP injection reduced startle habituation [CP × block interaction: *F*_(3, 123)_ = 5.8; *P* = 0.001] and this effect was particularly prominent in animals which had received young-adult chronic pre-treatment with CP [*F*_(3, 123)_ = 2.9; *P* = 0.039]. Thus, in female mice previously treated with saline, acute CP had no effect on startle habituation whereas in female mice previously treated with CP in young-adulthood, acute CP reduced startle habituation [*F*_(3, 60)_ = 6.4; *P* = 0.001]. Notably, none of these effects were different between wild-type and HET mice (Figure 7).

### [^3^H]CP55,940 binding

**Figure 8:**
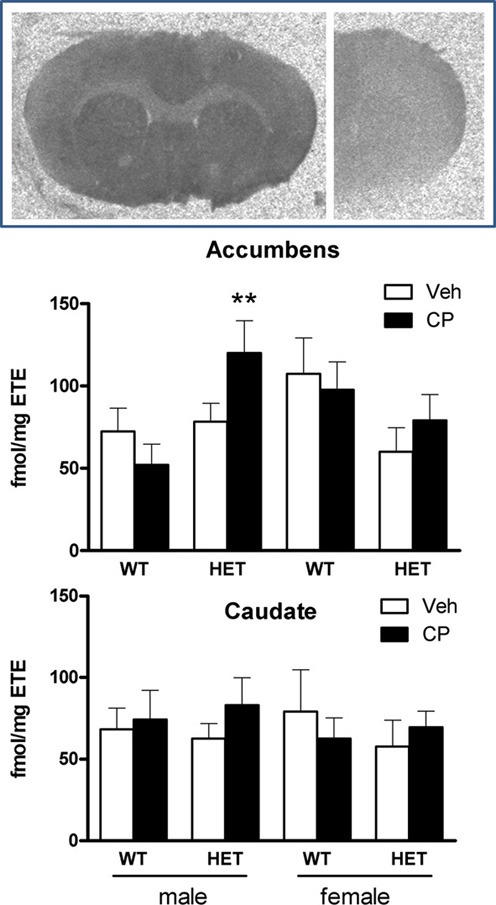
**Top: typical autoradiograms of [^3^H]CP55,940 binding in the mouse forebrain, showing total binding (left) and non-specific binding (right).** Specific binding densities were expressed as fmol/mg estimated tissue equivalent (ETE) in the nucleus accumbens (**Middle**) and caudate nucleus (**Bottom**) of male and female WT/veh, WT/CP, HET/veh, and HET/CP. Data are mean ± SEM. ^**^P < 0.05 for difference with BDNF HET mice which had received chronic vehicle injections.

Analysis of binding densities in the nucleus accumbens (Figure 8) revealed a significant effect of chronic young-adult CP treatment which was dependent on the genotype of the animals [*F*_(1, 40)_ = 5.8; *P* = 0.021] and there was also a sex × genotype interaction [*F*_(1, 40)_ = 5.7; *P* = 0.022]. Further analysis in wild-type mice showed that females had higher CP binding than males [*F*_(1, 22)_ = 5.6; *P* = 0.027] irrespective of prior CP exposure (Figure 8). In contrast, in HET mice, CP binding was significantly up-regulated [*F*_(1, 18)_ = 6.5; *P* = 0.020] and this effect appeared to be greatest in male mice (Figure 8) although the sex × CP term did not reach statistical significance. Indeed, analysis of data from male mice showed a significant CP × genotype interaction [*F*_(1, 20)_ = 4.7; *P* = 0.041] with no such effect in female mice, suggesting a selective up-regulation of cannabinoid binding density in the nucleus accumbens of male BDNF HET mice, but not wild-type mice (Figure 8). There were no sex differences, genotype differences or effects of young-adult CP treatment in the caudate nucleus (Figure 8).

## Discussion

The main findings of this study were that chronic young-adult treatment with a cannabinoid receptor agonist did not affect cognition or baseline PPI in adulthood in either wild-type or BDNF HET mice. However, acute injection of CP had differential effects on PPI depending on previous exposure to the drug as well as genotype and sex of the animals. Thus, male BDNF HET mice previously treated with CP showed a large increase in PPI upon acute CP treatment in adulthood whereas the other groups showed smaller or non-significant responses. This effect was paralleled by a significant increase of [^3^H]CP55,940 binding in the nucleus accumbens of male BDNF HET mice previously treated with CP.

In a previous study (Klug et al., 2012), we did not observe the lower baseline PPI seen in BDNF heterozygous mice in the present results. However, in that study, corticosterone was administered via the drinking water whereas CP55,940 was chronically injected in the present study. This chronic injection procedure may have induced or unmasked a lasting PPI deficit at baseline in BDNF HET mice. Furthermore, the PPI deficit was accompanied by an increase in startle amplitude which may have confounded the PPI result and was not seen in our previous study in BDNF HET mice (Klug et al., 2012). One explanation could be that BDNF HET mice show increased anxiety which could lead to an increase in startle amplitude. However, only one study found an increase in anxiety-like behavior (Chen et al., 2006) while most other studies did not find an anxiety-like phenotype on BDNF heterozygous mice (Montkowski and Holsboer, 1997; Macqueen et al., 2001; Chourbaji et al., 2004; Ibarguen-Vargas et al., 2009). Another explanation for the enhanced startle amplitude could be the difference in bodyweight between the genotypes. HET mice had significantly higher bodyweights than wild-type mice and this could at least in part account for greater startle amplitudes.

Chronic CP treatment during young adulthood did not lead to a deficit in baseline PPI in adulthood which is similar to what we observed in a recent study in rats (Klug and Van Den Buuse, 2012). Acute injection of CP increased PPI and in male mice this was predominantly in HETs previously treated in young-adulthood with CP. In another study, acute injections of CP was shown to cause a decrease of PPI in wild-type mice but this effect disappeared when the mice were treated chronically (Boucher et al., 2011). It is possible that CP can influence PPI in both directions which would be in accordance with studies in rats where some studies found a decrease in PPI (Martin et al., 2003; Malone and Taylor, 2006) while others reported increased PPI (Stanley-Cary et al., 2002). This may be due to methodological differences between the studies as it has been shown that different solvent agents can influence the behavioral outcome (Stanley-Cary et al., 2002). However, Boucher et al. used a similar method to ours to dissolve their CP and showed decreased PPI in wild-type mice after acute treatment (Boucher et al., 2011). The group of BDNF HET male mice previously exposed to CP which responded with an increase in PPI also had the lowest PPI score of all male groups at baseline. After acute treatment with CP, PPI levels were indistinguishable in this group from the other groups. This seems to be comparable to cannabis self-medication which has been suggested in people with schizophrenia as a means to alleviate some of their symptoms (Phillips and Johnson, 2001; Schofield et al., 2006). It is possible that CB1 receptor stimulation is effective in reversing disruptions of PPI or other schizophrenia-relevant behaviors, independent of its effect on baseline behaviors. For example, similar to our present results, acute treatment with a CB1 receptor agonist reversed PPI deficits in psychosocially stressed mice (Brzozka et al., 2011) and phencyclidine-treated rats (Spano et al., 2010). On the other hand, in an isolation-rearing stress model, Malone and Taylor (2006) observed a decrease of PPI after acute treatment with THC, which had no effect in normally-housed control (Malone and Taylor, 2006). Future studies could focus on the effects of cannabinoid receptor stimulation in other models of genetic or environmentally-induced PPI disruptions. For example, recently it was shown that BDNF treatment produced long-lasting reversal of PPI deficits in DBA mice (Naumenko et al., 2013) and it would be of interest to study the acute and chronic effects of cannabinoid receptor stimulation in this strain.

Some caution is needed when interpreting the PPI results as acute treatment with CP reduced startle amplitudes which may have confounded PPI. The reduction in startle response is most likely related to adverse effects of the cannabinoid agonist that include hypoactivity and catalepsy (Chaperon and Thiebot, 1999). Other studies have also failed to detect cannabinoid-induced effects in PPI that were not accompanied by impairment in startle reflex (Nagai et al., 2006; Boucher et al., 2011). For example, Martin et al. only found a PPI deficit when rats were treated with the highest dose of CP which also decreased startle amplitudes. When the animals were treated with a lower dose, no effects on startle reactivity were observed but the previously observed PPI deficit had vanished as well, showing that CP treatment does not impair PPI without affecting startle (Martin et al., 2003). This raises the possibility that the effects of CP on PPI are not a real impairment in sensorimotor gating (Swerdlow et al., 2000). Further studies should be considered to see whether a smaller dose of CP would lead to an enhancement in PPI without affecting startle amplitudes. It noteworthy, though that CP only increased PPI in some groups while it reduced startle in all groups, which would suggest that the presently observed selective PPI changes are independent of the effect of CP on startle.

The selective increase in the effect of acute CP in male BDNF HET mice previously treated with the drug, was paralleled by a selective increase in CP binding density in the nucleus accumbens in these mice. The nucleus accumbens plays an important role in PPI regulation (Shilling et al., 2008; Roncada et al., 2009). CB1 receptors are present on fast-spiking interneurons in this nucleus which form inhibitory GABAergic synapses with medium-spiny interneurons and modulate its activity (Winters et al., 2012). These neurons can also modulate behavioral effects of dopaminergic psychotropic drugs (Corbille et al., 2007) and the selective up-regulation of CP binding density in the present study may explain the enhanced effect of acute treatment with the drug on PPI. Similar to the present results, previous studies have found that chronic cannabinoid receptor agonist treatment could influence CB1 receptor levels and function even long after treatment had finished (Zamberletti et al., 2012). However, it is important to note that the single-point agonist binding method used here does not distinguish between possible changes in Kd or Bmax of the receptor. Therefore, future, more elaborate binding studies are needed to extend the present results and ascertain the molecular basis of the changes in [^3^H]-CP55,940 binding density. These further studies could also include other CB1 compounds as displacing agents.

Another possible mechanism involved in the present results could be the effect of acute and chronic CP treatment on BDNF levels. Previous animal studies have shown that THC increases BDNF gene expression acutely (Derkinderen et al., 2003) and after chronic administration (Butovsky et al., 2005). In human studies, THC increased serum BDNF levels in healthy controls, but not in chronic cannabis users (D'souza et al., 2009). It would be of interest to assess if young-adult CP treatment, as used in the present study, induced long-term reversal of the reduced BDNF levels seen in BDNF HET mice (Hill and Van Den Buuse, 2011). This might also explain the sex differences observed in the present study, as young-adult developmental changes in BDNF levels showed marked male-female differences in normal mice (Hill et al., 2012) and sex-specific alterations in BDNF signaling were found in BDNF HET mice (Hill and Van Den Buuse, 2011).

In addition to its effect on PPI, chronic CP treatment also affected locomotor activity as was expected from previous studies (McGregor et al., 1996; Boucher et al., 2011; Llorente-Berzal et al., 2011). Interestingly, male mice treated with CP during young-adulthood showed reduced locomotor activity even 2 weeks after treatment had ceased. This is similar to what we observed in rats (Klug and Van Den Buuse, 2012) and it seems that male animals are more prone toward the long-term effects that CP can exert on locomotor activity. There were other, more subtle and sex-dependent differences in locomotor activity between the genotypes at 6 and 9 weeks. However, none of these effects were seen in adulthood at 11 weeks of age similar to what has been observed in previous studies (Chourbaji et al., 2004; Saylor and McGinty, 2008; Klug and Van Den Buuse, 2012).

No effects of either genotype, cannabinoid treatment or the combination of the two were observed in the Y-maze tasks. All animals spent more time in the novel arm which is an indicator for intact short-term spatial memory (Dellu et al., 2000). In a previous study, we observed marked deficits in Y-maze performance in male BDNF HET mice treated chronically with corticosterone in young adulthood (Klug et al., 2012). These differential results show that various young-adult “second hits” can have markedly different outcome in adulthood, in this case corticosterone exposure compared to cannabinoid treatment. Novel object recognition was impaired in BDNF HET mice which has been reported before (Seoane et al., 2011). However, similar to Y-maze performance, there was no effect of chronic CP treatment, strengthening the selectivity of its action on PPI. Thus, although previous studies have shown involvement of both the endocannabinoid system and BDNF in memory formation and consolidation (Papaleo et al., 2011; Panlilio et al., 2012; De Bitencourt et al., 2013; Wright et al., 2013), in our protocol including chronic CP treatment followed by a 2-week washout, there were no such effects. Future studies could include the acute effects of cannabinoid receptor stimulation on memory function in BDNF HET mice and controls after chronic pre-treatment with CP, similar to the PPI studies presented here.

In conclusion, this study shows that chronic cannabinoid treatment during young adulthood in mice does not lead to major long-lasting behavioral effects at baseline in adulthood. However, it appears that chronic cannabinoid treatment in male mice with a BDNF deficiency makes the animals more sensitive toward the acute effects of cannabinoid receptor stimulation later on in life. This could have implications for the effects of cannabis abuse in humans in a subset of individuals with low BDNF signaling.

### Conflict of interest statement

The authors declare that the research was conducted in the absence of any commercial or financial relationships that could be construed as a potential conflict of interest.
